# Supra-Epiglottic Upper Airway Volume in Elderly Patients with Obstructive Sleep Apnea Hypopnea Syndrome

**DOI:** 10.1371/journal.pone.0157720

**Published:** 2016-06-23

**Authors:** Claire Boutet, Syad Abdirahman Mohamed Moussa, Sébastien Celle, Bernard Laurent, Jean-Claude Barthélémy, Fabrice-Guy Barral, Frédéric Roche

**Affiliations:** 1 Inserm U1059, Univ Lyon, Department of Radiology, Pole IMOFON, CHU of Saint-Etienne, Saint-Etienne, France; 2 Department of Radiology, Pole IMOFON, CHU of Saint-Etienne, Saint-Etienne, France; 3 EA 4607 SNA EPIS, Clinical Physiology and VISAS Center, Pole NOL, CHU and Faculty of Medicine of Saint-Etienne, University Jean Monnet Saint-Etienne, COMUE Lyon Saint-Etienne, France; 4 Neurology/Neuropsychology, Center Memory of Resources and Research Unit, Pain Center, North Saint-Etienne University Hospital Center, Central Integration of Pain, Lyon Neuroscience Research Center, Bron, France; National Institute for Viral Disease Control and Prevention, CDC, China, CHINA

## Abstract

**Objective:**

Small upper airway measurements areas and high body mass index are recognized risk factors for obstructive sleep apnea syndrome (OSAS) in non-elderly populations; however, there is limited information regarding elderly patients. We evaluated whether upper airway volume is associated with OSAS and OSAS treated with continuous positive airway pressure (CPAP) treatment and whether BMI is correlated with upper airway volume and measurements in elderly subjects.

**Methods:**

In 60 volunteers aged 75.58±0.9 years: 20 OSAS, 20 OSAS chronically treated with CPAP, and 20 controls, semi-automatic segmentation, retropalatal distance and transverse diameter of the supra-epiglottic upper airway were evaluated using 3DT1-weighted magnetic resonance imaging. Anteroposterior to transverse diameter ratio was defined as retropalatar diameter/transverse diameter.

**Results:**

There were no significant differences in supra-epiglottic upper airway volume between OSAS, CPAP treated patients, and controls. There were significant differences in retropalatal distance and anteroposterior to transverse diameter ratio between OSAS, CPAP treated patients, and controls (*P* = 0.008 and *P*<0.0001 respectively). There was a significant correlation between body mass index and retropalatal distance (*P*<0.05) but not with supra-epiglottic upper airway volume.

**Conclusion:**

In elderly subjects, OSAS and body mass index are not associated with changes in supra-epiglottic upper airway volume but are associated with modification of pharynx shape.

## Introduction

Obstructive sleep apnea syndrome (OSAS) is a type of sleep-disordered breathing characterized by repetitive complete or partial upper airway obstruction during sleep [1]. This common disorder is especially prevalent, occurring in approximately 24% of middle-aged and elderly adults [2, 3]. OSAS has been associated with nighttime hypertension [4], reduced renal function [5], cognitive impairment [6], and nocturia in the elderly [7, 8]. Unlike younger patients, OSAS is less associated with developing cardiovascular disease or stroke in elderly subjects [9, 10]. However, for individuals with preexisting cardiovascular disease, frequent hypoxic events such as apneas are associated with mortality [11, 12]. In addition, it’s been shown that with older men, sleep-disordered breathing was not associated with daytime sleepiness or other sleep-related symptoms in contrast to studies with younger patients, indicating that the functional consequences of sleep disorders may differ in the elderly [13].

It is evident that there are natural changes in upper airway anatomy as an individual ages, and these changes can have a profound impact on how we evaluate and address OSAS in the elderly. A recent study using three-dimensional (3D) cone-beam computed tomography (CT) to evaluate the normal changes in the upper airway over time found that upper airway length and volume increase until approximately age 20, plateau until age 50, and then rapidly decrease [14]. In contrast, two-dimensional (2D) CT and lateral cephalometry studies found that elderly snorers and OSAS patients (>63-years-old) have larger upper airway volumes than younger ones [15]. In addition, another study found that general respiratory disturbances during sleep increase significantly as a person ages, even in asymptomatic individuals [16]. These discrepancies highlight the need to conduct more studies in order to identify the factors that occur naturally with age and those that contribute to OSAS in elderly patients. In particular, the upper airway morphological changes in response to continuous positive airway pressure (CPAP) treatment have never been evaluated in an elderly population.

Many studies have evaluated apneic obstruction by looking at upper airway measurements and volumes using techniques such as lateral cephalometry, CT [15, 17], and magnetic resonance imaging (MRI) [18–21]. Lateral cephalometry and cone-beam CT allow three-dimensional (3D) reconstructions; however, they subject the patient to significant amounts of radiation and are often collected in an upright position, which does not reflect the airway dimensions that are present when sleeping. An MRI is a viable alternative, because there is no radiation and the measurements are taken in a supine position, which models more accurately the upper airway configuration during sleep. Several studies have used MRI to assess upper airway measurements; however, these studies primarily used linear [19] or surface measurements [20, 21], and they did not stratify their subjects by age. One study of OSAS patients after stroke found that the retropalatal distance is smaller in stroke patients with OSAS than those without OSAS [19]. One MRI study identified an elliptic pharynx shape with a longer transverse axis in healthy subjects, and a circular or less elliptic shape caused by a transverse axis decrease in OSAS subjects [21].

While 2D analyses are valuable, a significant amount of morphological information outside of the immediate area of interest is lost. However, technology has improved such that routine, semi-automatic 3D-MRI reconstructions can feasibly be performed. Currently, studies using 3D-MRI reconstructions of upper airway dimensions to evaluate OSAS are limited and primarily methodological [20, 22–24]. This technique provides a more comprehensive picture of upper airway changes in OSAS patients. Therefore, we used a semi-automated 3D-MRI reconstruction algorithm to evaluate the upper airway in this study.

Given the need for data regarding upper airway morphology in elderly OSAS patients and the availability of semi-automatic 3D-MRI reconstruction software, we investigated the supra-epiglottic upper airway volume of elderly OSAS patients, before and after long term CPAP treatment use, and compared them with age- and sex-matched control subjects.

## Materials and Methods

### Population

The participants in this study were recruited from an existing prospective clinical trial, the PROgnostic indicator OF cardiovascular and cerebrovascular events (PROOF), which aimed to assess the influence of autonomic nervous system activity on cardiovascular and cerebrovascular morbidity and mortality. The initial population cohort consisted of 1,011 retired volunteers aged 65-years-old (609 women, 402 men) that had enrolled in 2001 by random selection from the city of Saint-Etienne, France electoral list. Exclusion criteria included the following: previous myocardial infarction; arrhythmia; cardiac pacemaker; stroke; neurological or psychiatric disease; insulin-dependent diabetes mellitus; a cerebral MRI suggesting a neurological disease or dementia; residing in an institution; or moving out of the city within the next 2 years. According to the French government’s National Institute for Statistics and Economics Studies, the 1,011 participants were representative of the French population aged 65-years-old in terms of sex and educational level [25].

An ancillary study addressing the association between OSAS, assessed by ambulatory polygraphic recording, and cardiovascular and cerebrovascular morbidity during a 7-year follow-up was proposed to the participants (the Synapse study). The criteria for inclusion in the Synapse study was as follows: absence of a previous myocardial infarction, stroke, or atrial fibrillation; absence of previous OSAS diagnosis or treatment; and willingness to undergo polygraphy, 24-h blood pressure and heart rate monitoring, and blood sample collection. A total of 854 subjects (58.5% women) meeting the inclusion criteria were enrolled in the Synapse study. All subjects underwent a clinical assessment including a demographic characteristics questionnaire, medical history and medication collection, and an Epworth Sleepiness Scale (ESS) evaluation. Hypopnea was defined as a 50% or greater reduction in the airflow from baseline lasting at least 10 sec that was associated with at least 3% oxygen desaturation. Apnea was defined as the absence of airflow on the nasal cannula lasting at least 10 sec. The AHI was established as the ratio of the number of apneas and hypopneas recorded per hour. Oxyhemoglobin desaturation index (ODI) was defined as the number of episodes of oxygen desaturation per hour of recording time during which the SpO2 decreased by 3% or more. The presence of OSAS was defined as an apnea-hypopnea index (AHI) ≥ 15 [16]. At baseline, AHI > 15 was found in 54% of subjects with 18% having an AHI > 30. Subjects with diagnosis of OSAS were referred to their physician and treated with CPAP if necessary. The data used in the present study were gathered from the third examination of the PROOF study (May 2009– February 2012).

We randomly selected 20 OSAS subjects chronically treated with CPAP (t-OSAS; 19 men and 1 woman; duration of CPAP treatment, 1.5 years ± 0.3), and then 20 OSAS subjects (15 men and 5 women) and 20 Control Subjects (17 men, 3 women, AHI<15/h, no snoring complain and ESS score <10) matched for age and sex from the Synapse cohort. Patients of CPAP treatment (fixed mean pressure 11± 2 cmH2O) demonstrated a mean night use of 5h40 ± 0h25 of their device with normalization of their excessive daytime sleepiness in all cases.

The University Hospital and the Institutional Review Board–Independent Ethics Committee (CCPRB Rhône-Alpes Loire) approved the PROOF study. The National Committee for Information and Liberty gave consent for data collection. All subjects provided written informed consent for study participation.

### Anthropometric measurements

BMI was calculated as weight/height squared (kg/m^2^) [26]. Neck circumference (NC), in cm, was measured at the middle of the neck between the mid-cervical spine and the mid-anterior neck 0.5 cm below the laryngeal prominence.

### MRI acquisition

All MRIs were acquired on the same whole-body 1.5 T scanner (Magnetom Avento, Siemens Healthcare, Erlangen, Germany) with a 12-channel head coil. The device was located in the Department of Radiology at the University Hospital of Saint-Etienne. The patients were imaged in the supine position with the Frankfort plane oriented perpendicular to horizontal. Patients were instructed to breathe through their nose during the imaging. We removed CPAP to perform MRI in OSAS with CPAP treatment. All scans were conducted by a board-certified MRI technologist using a standard protocol. The acquisition protocol was a 3D T1-weighted magnetization-prepared rapid-gradient-echo (MP-RAGE) sequence with the following acquisition parameters sequence: isotropic resolution of 1 × 1 × 1mm^3^; repetition time, 2060 ms; echo time, 3.23 ms; inversion time, 1100 ms; acquisition matrix 256 × 256; number of slices, 176. Data were collected from May 2009 to February 2012.

### Semi-automated segmentation of the supra-epiglottic upper airway

Supra-epiglottic upper airway segmentation was carried out on the 3D T1 MRI sequences with a semi-automatic algorithm using the PACS software (Carestream Health, Rochester, NY). The supra-epiglottic upper airway volume (cm^3^) for each subject was obtained through semi-automatic segmentation by increasing the signal intensity. The limits of the segmented regions were defined on sagittal sections with primary references in the axial and coronal sections to ensure 3D consistency. The superior border of the supra-epiglottic upper airway was set at the lower faces of the sphenoïdal and basilar occipital bones, and the inferior border was defined at the base of the epiglottis. Anterior and posterior borders were defined using the anterior and posterior pharyngeal soft tissues. Each segmentation was performed three times, and the average of the three segmented volumes was used in this work. The total segmentation time was 10 min/subject. Segmentation quality was controlled by two observers that were blind to the clinical data and diagnosis.

### Linear measurements

For comparison with volumetric measures, we also performed manual linear measurements for retropalatal diameter (the horizontal distance between the posterior pharyngeal wall and the soft palate at the level of the hard palate) on the middle sagittal section and transverse diameter on axial section at the level of hard palate, perpendicular to the retropalatar diameter. The anteroposterior to transverse diameter ratio was defined as retropalatar diameter/transverse diameter. Linear measurements were made using the PACS software (Carestream Health, Rochester, NY) by the same rater (CB), who was blind to both segmentation and clinical data and diagnosis.

### Statistical analysis

Clinical characteristic statistical comparisons of the control, OSAS, and t-OSAS subjects were performed using analysis of variance (ANOVA) for age, using Chi-square for gender, and using Kruskal–Wallis for BMI, ESS, linear measurements and supra-epiglottic upper airway volume. Post-hoc analyses were performed with a Mann–Whitney U test for linear measurements and supra-epiglottic upper airway volume. The strength of the relationship between the supra-epiglottic upper airway volume and BMI and between the linear measurements and BMI was estimated using the Pearson correlation coefficient. The significance threshold was defined as p<0.05. The results are presented as means ± standard deviation (SD). Statistical analyses were performed using MedCalc for Windows, version 13.1.2 (MedCalc Software, Mariakerke, Belgium).

## Results

### Characteristics of the study population

Demographic data are given in Table 1. The three groups did not differ for age (F_2_^57^ = 1.96, p = 0.15), gender (χ² = 3.14, two degrees of freedom, p = 0.21) and BMI (H_2_^60^ = 2.91; p = 0.23).

**Table 1:** Demographic and clinical data.

	Control Subjects	t-OSAS	OSAS
**Number of subjects**	20	20	20
**Women/men**	3/17	1/19	5/15
**Age [years]**	75.06 **±** 0.9	75.27 **±** 0.9	75.6 **±** 0.9
**BMI [kg/m²]**	27.61 ± 2.8	28.61 ± 3.5	29.37 ± 3.4
**Neck circumference [cm]**	37.47 ± 3.7	40.92 ± 3.5	40.1 ± 4.2
**ESS**	4.6 ± 3.4	8.6 ± 3.8	6.5 ± 3.6
**AHI [n/h]**	6.79 ± 3.6	50.1 ± 16.2	30.75 ± 18.8
**ODI [n/h]**	0.9 ±0.8	31.6 ±10.3	17.9 ±7.0

Data are given as mean ± standard deviation. Abbreviations: OSAS, obstructive sleep apnea syndrome; t-OSAS, obstructive sleep apnea syndrome treated with continuous positive airway pressure; BMI, Body Mass Index; ESS, Epworth Sleepiness Scale evaluation; AHI, apnea plus hypopnea index; ODI, Oxyhemoglobin Desaturation Index. For t-OSAS subjects, baseline ESS, AHI and ODI are given.

Baseline ESS differed significantly between the three groups (H_2_^60^ = 10.62; p = 0.005), with a significant difference between control and t-OSAS subjects (p<0.05). The neck circumference was significantly different between the three groups (H_2_^60^ = 5.92; p = 0.05), with a significant difference between control and t-OSAS subjects (p<0.05). A significant correlation between BMI and neck circumference (*r* = 0.31; p<0.05) was also found.

### Automated segmentation of the supra-epiglottic upper airway

**Fig 1:**
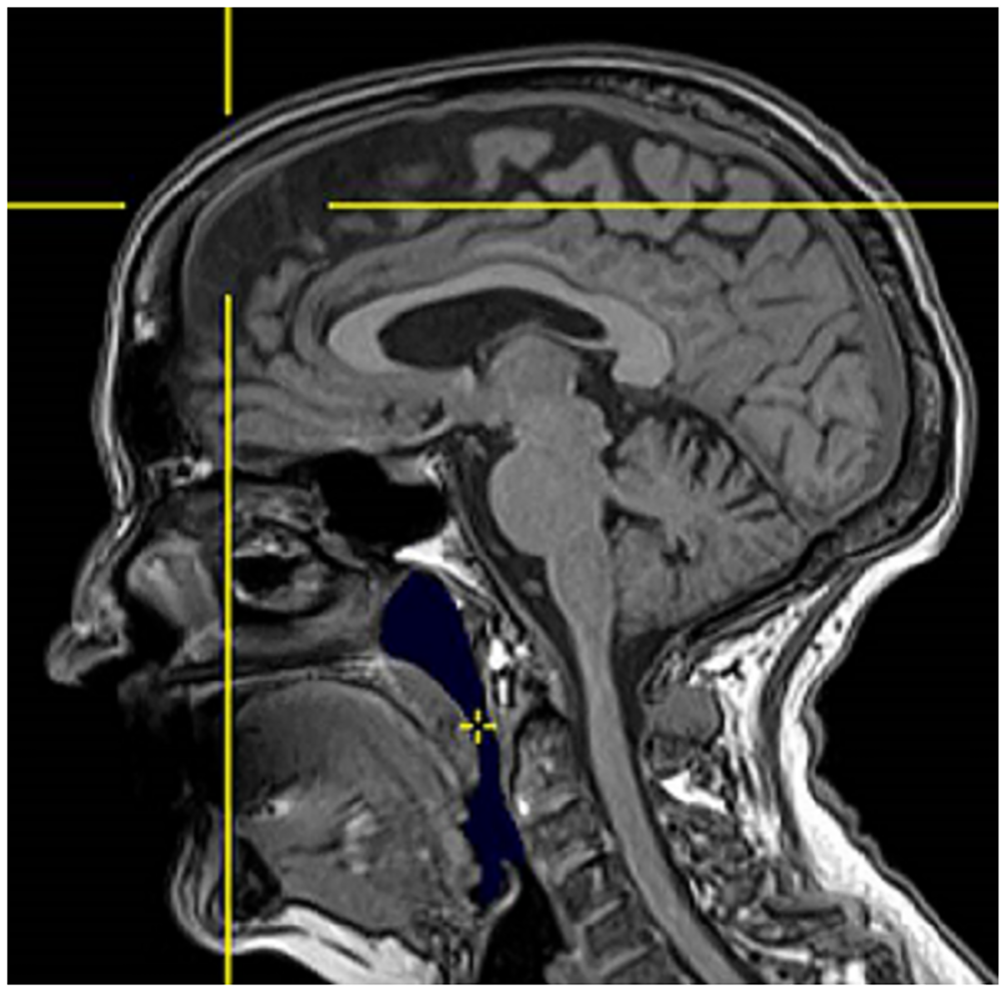
Sagittal slice of a three-dimensional T1-weighted magnetic resonance image acquisition in a single control subject. The semi-automatic segmented volume is shown in blue. The cross indicates the initial point of growth for the volume calculation.

Example of semi-automatic segmented volume is presented in Fig 1.

Volume data are given in Table 2. The supra-epiglottic upper airway volumes did not differ between the three groups (H_2_^60^ = 3.57; p = 0.17). There was no significant correlation between BMI or neck circumference and supra-epiglottic upper airway volume (p = 0.51 and p = 0.14 respectively).

**Table 2:** Supra-epiglottic upper airway measurement data.

	Control Subjects	t-OSAS	OSAS
**Airway volume [cm**^**3**^**]**	11.64 ± 3.5	11.2 ± 3.6	13.29 ± 3.8
**Retropalatal distance [mm]**	19.19 ± 5.14	17.18 ± 4.9	14.34 ± 3.5*
**Transverse diameter [mm]**	21.69 ± 4.9	24.69 ± 4.7	23.84 ± 3.1
**AP/T diameter ratio**	1.18 ± 0.3	1.51 ± 0.4*	1.77 ± 0.5*

Data are given as mean ± standard deviation. Abbreviations: OSAS, obstructive sleep apnea syndrome; t-OSAS, obstructive sleep apnea syndrome treated with continuous positive airway pressure; AP/T diameter ratio, anteroposterior to transverse diameter ratio.

* Indicates a significant difference between OSAS or t-OSAS and control subjects (Mann–Whitney U test, p<0.05).

### Linear measurements

Linear measurements data are given in Table 2. The retropalatal distance was significantly different between the three groups (H_2_^60^ = 9.63; p<0.05), with a significant difference emerging between control and OSAS subjects (p<0.05). The three groups did not differ for transverse diameter (H_2_^60^ = 3.11; p = 0.21). The anteroposterior to transverse diameter ratio were significantly different between the three groups (H_2_^60^ = 18.82; p<0.0001), with a significant difference between control and t-OSAS patients (p<0.05), and between control and OSAS subjects (p<0.0001). There was no significant correlation between neck circumference and retropalatal distance (p = 0.34), transverse diameter (p = 0.22), or anteroposterior to transverse diameter ratio (p = 0.34). BMI correlated significantly with both retropalatal distance (*r* = -0.32; p<0.05) and transverse diameter (*r* = -0.26; p<0.05).

## Discussion

In this study we investigated the changes in supra-epiglottic upper airway volume between elderly control, OSAS, and t-OSAS subjects using 3D MRI. The results demonstrated that for elderly subjects, supra-epiglottic upper airway volume does not differ between normal and OSAS individuals, whereas the retropalatal distance increased and the anteroposterior to transverse diameter ratio decreased in OSAS patients versus controls. The linear results are similar to other studies on younger subjects that had found reduced upper airway measurements in OSAS patients [15, 21, 27, 28]. Rodenstein et al. [21] found that the soft palate area and the anteroposterior to transverse diameter ratios were larger in OSAS patients versus controls and snorers. Meyer et al. [15] demonstrated that soft palate length and hypopharyngeal area were only significantly larger in OSAS patients compared to snorers if the subjects were less than 52-years-old. An ultrafast MRI study of control subjects, awake OSAS patients, and sleeping OSAS patients found that the minimal velopharynx area was significantly smaller in OSAS patients than in controls, and that the area further decreased when the patients were asleep [27]. Furthermore, one CT study had shown that the uvular cross-sectional area was significantly more narrow in OSAS patients upon expiration [28]. However, all of these results were collected using 2D methods and focused on specific upper airway measurements that do not necessarily translate to overall decreased upper airway volume. A study that used 3D multi-detector CT to assess upper airway changes based on OSAS severity found no significant differences in upper airway volume between controls and mild/moderate or severe OSAS patients, despite the mean ages of the subjects ranging from 36–44-years-old [29]. The data in younger subjects is consistent with our results in elderly patients, indicating that OSAS may not depend exclusively on reduced upper airway volumes. In that study, the OSAS was attributed to an increase in the vertical length between the hard palate and hyoid [29]. Clearly, there are a number of anatomical factors that contribute to upper airway volume, and further studies using 3D reconstruction are needed to better understand the changes associated with age and OSAS.

We found no difference in the upper airway volume and linear measurement of t-OSAS patients compared with OSAS; this is significantly different from previous reports of upper airway measurements in CPAP-treated patients. One study on the short- and long-term effects of CPAP on the upper airway using acoustic pharyngometry demonstrated that the oropharyngeal junction, maximum pharyngeal area, and mean pharyngeal area had significantly increased, after both 1-week and 6-months post-treatment initiation [30]. They also demonstrated that a 1-week cessation of CPAP treatment commensurately reduced these values. However, this study noted a change in upper airway anatomy within the same patients over time; because this was a prospective study, it would therefore be much more likely to find a difference than our cross sectional cohort. Mortimore et al. [31] showed that the posterior airway space in the supine position significantly increased following 3 months of CPAP treatment and that the change in airway space was correlated with CPAP compliance. The mean age in these studies was 55- and 49-years-old, respectively. According to these data, there should be a difference in upper airway volume, but this was not seen in our elderly patient data, where t-OSAS and OSAS presented a different baseline severity. These studies provide several potential hypotheses regarding the mechanisms for the upper airway changes following CPAP treatment, including reduced upper airway edema, improved upper airway dilator muscle function [30, 31], reduced airway collapsibility, and reduced fatigue of upper airway muscles [31]. It is worth noting that many of these hypotheses center on improved muscle function. Given the propensity for muscle hypotonia in the elderly [32], it seems likely that the pharyngeal muscles may not recover as readily as those in younger individuals. Thus, CPAP treatment may not produce a profound increase in upper airway volume in the elderly.

Another interesting result was the lack of correlation between BMI and NC and upper airway volumes. BMI is a well-known factor contributing to OSAS severity and is one of the strongest predictors of sleep-disordered breathing [33]. Of the various mechanisms hypothesized to link obesity with OSAS, changes in upper airway structures are a popular theory. Pharyngeal loading of subcutaneous fat and infiltration of periluminal fat deposits can narrow the airway and fatigue muscles. These fat deposits can also cause alterations in pharyngeal shape [33], consistent with the correlation between BMI and linear measurement that we found. In addition, several longitudinal studies have shown that small changes in weight can significantly alter the AHI and respiratory-disturbance index [34, 35]. However, these data do not necessarily apply to older patients. In a study by Meyer et al. [15] comparing OSAS patients and snorers, BMI only correlated with AHI if the subjects were less than 52-years-old and when the patients were over 63-years-old, the correlation disappeared. These data are very consistent with our observations. At this time, it is unclear why this phenomenon occurs. Meyer hypothesized that it may be related to increase upper airway volume or unidentified confounding factors in elderly patients [15]. In our cohort, the upper airway volume did not increase; therefore, other causal factors must be in play, such as alterations in pharyngeal shape.

There were a number of limitations to this study. First, due to the imaging time, the airway volume collected is the mean value rather than being associated with a specific breathing phase. In addition, these data were collected on subjects who were awake. The ultrafast MRI study described above showed that the difference between healthy and OSAS subjects was observed during expiration when the minimal velopharynx area was smallest, and the smallest area occurred in sleeping subjects [27]. Thus, the differences in airway volume may not be as evident when averaged across multiple breathing cycles in subjects who were awake. Secondly, our OSAS subjects had little to no symptoms and their OSAS was discovered during the study, which is different from the OSAS populations usually published. Moreover, in the elderly, OSAS is more often related to REM sleep which can explain the main role of muscular hypotonia compared to the anatomical alternatives of volumes.

In conclusion, our results have shown that there is no difference in supra-epiglottic upper airway volume between elderly control subjects, OSAS patients, and OSAS patients undergoing long term CPAP treatment, with a significant difference in retropalatal distance and in the anteroposterior to transverse diameter ratio between elderly control subjects and OSAS patients. These data support the theory that the mechanism of OSAS may be caused by morphological differences rather than overall volume loss. Our results also show that BMI is not correlated to upper airway volumes in elderly subjects, which is consistent with previous observations. Surprisingly, we found that CPAP treatment may not have lasting effects on upper airway volume in the elderly, which conflicts with results seen in younger subjects; however, our study was not a longitudinal study. Future studies looking at pharyngeal anatomical features are needed to fully clarify the mechanisms behind OSAS in the elderly. Overall, these data support the theory that sleep breathing characteristics change as an individual ages, and as such, elderly OSAS patients may require different diagnostic criteria and treatment strategies.

## Abbreviations

AHI: apnea-hypopnea index
ANOVA: analysis of variance
BMI: body mass index
CPAP: continuous positive airway pressure
CT: computed tomography
ESS: Epworth Sleepiness Scale
NC: neck circumference
MRI: magnetic resonance imaging
OSAS: obstructive sleep apnea syndrome
PROOF: PROgnostic indicator OF cardiovascular and cerebrovascular events Trial
SD: standard deviation
t-OSAS: obstructive sleep apnea syndrome treated with continuous positive airway pressure
2D: two-dimensional
3D: three-dimensional
